# Editorial: Phytochemicals in ornamental plants: Synthesis, nutraceutical prospects and applied focus—Women in plant science series

**DOI:** 10.3389/fpls.2022.1016968

**Published:** 2022-09-05

**Authors:** Valentina Schmitzer, Wee Sim Choo, Andreja Urbanek Krajnc

**Affiliations:** ^1^Biotechnical Faculty, Department of Landscape Architecture, University of Ljubljana, Ljubljana, Slovenia; ^2^School of Science, Monash University Malaysia, Subang Jaya, Malaysia; ^3^Faculty of Agriculture and Life Sciences, University of Maribor, Maribor, Slovenia

**Keywords:** phytochemicals, ornamental plants, *Brassica oleracea*, *Skimmia anquetilia*, *Clitoria ternatea*

Increased interest for potent nutraceuticals has prompted the research and breeding community to look beyond conventional plant sources and examine the potential of ornamental plants as they contain many valuable metabolites. Traditionally, wild flowering and fruiting plants such as species of the *Cornus, Rosa* and *Sambucus* genus were included in human diets and new sources are now investigated in contemporary studies. Lesser-known wild flowering perennials and woody plants were used locally and the benefits of their components are now studied. Flowers, leaves, fruits and other parts of ornamentals are not only inspiring due to their visual and landscaping traits but can be explored and exploited further—for their phytochemical composition linked to various applied functions. These include functional foods, food additives and colorants, allelochemicals, plant growth stimulants, natural products for pest and disease control among others. Plant-based and natural supplements are gaining importance; increasing the synthesis of favorable metabolites in ornamental plants will be an important focus in the future.

The aim of this research focus was to expand data on all segments of ornamental plant phytochemistry—from gene expression and biosynthetic steps to metabolomics and accumulation of specific compounds with different functions. The aim was to highlight the current state of research in phytonutrient synthesis of flowers, fruits, and other parts of ornamental plants and to recommend specific wild and cultivated plants as sources of phytonutrients for applied purposes. A large proportion of the population (80%—WHO from developing and underdeveloped countries) still depends on natural products for their wellbeing, so ornamental plants have great potential for the future. Another aim was to present global views on this topic and encourage women, minorities and young scientists to contribute to this Research Topic.

The current Research Topic includes two review articles and two original research articles focusing on (i) phytochemical screening covering all steps from single compound biosynthesis, extraction, stability, antioxidant activity, antibacterial activity to ethnobotanical use and future applications, and (ii) transcriptome analysis of leaf colouration-related genes and pathways that have great breeding and agronomic importance for nutritionally important plant species.

The article by Zhou et al. focuses on the analysis of the transcriptome and pigment content of green and white leaf tissue of ornamental cabbage, by evaluating three different phenotypes. The study found that the green and white colouration of ornamental cabbage leaves is caused by the combined effects on “porphyrin and chlorophyll metabolism” and “carotenoid biosynthesis,” chloroplast biogenesis as well as photosynthesis. The authors discovered several candidate genes for “photosynthesis,” “photosynthesis-antenna proteins,” “carbon fixation” as well as HSPs that might be involved in the development of leaf color. By observing different expression trends in certain carotenoid biosynthesis genes, the authors have also highlighted the possible regulatory effect of abscisic acid in color formation. A thorough knowledge of pigment synthesis is not only important for ornamental plants, but also has great breeding and agronomic significance for nutritionally important plant species.

The review article by Nabi et al. (A) highlights the past and current knowledge on the medicinal plant *Skimmia anquetilia*, which has sixteen different vernacular names in India and has long been used in the Ayurvedic and Unani systems of traditional medicine. It is known for the efficacy of its bioactive molecules in a range of health-related conditions and diseases, exhibiting antibacterial, antioxidant, anti-inflammatory, anti-nutritional and anticancer activities. The authors highlight the wide arsenal of individual alkanes, alkanes, alkenes, coumarins, carboxylic acids, fatty acids and their esters, terpenes and phenols and their pharmaceutical and nutritional effects. The authors provide a critical evaluation of the historical perspective on the use of plants, helping to recover their potential as a source for drug development of predominant molecules.

In an experimental study, Nabi et al. (B) tested antibacterial activity of *Skimmia anquetilia* root extracts against multidrug-resistant strains of *Pseudomonas aeruginosa, Escherichia coli, Klebsiella pneumoniae, Salmonella typhi*, and *Staphylococcus aureus*. As these bacteria are responsible for several healthcare acquired infections the potential of natural drugs for their inhibition should be thoroughly investigated. The authors identified diverse phytoconstituents in root extracts, dependant on the solvent solution. The predominant compounds were 5, 10-pentadecadien-1-ol and n-hexadecanoic acid in n-hexane extract, 5,10-pentadecadien-1-ol and 1-hexyl-2-nitrocyclohexane in ethyl acetate extract, and 1-hexyl-2-nitrocyclohexane and cis,cis,cis-7,10,13-hexadecatrienal in methanol extract. Ethyl acetate extract of *Skimmia anquetilia* demonstrated significant antibacterial activity against a *Staphylococcus aureus* strain. These results support the invention of plant-based medicines using *S. anquetilia*, which may lead to the development of novel remedies against drug-resistant microbial infections.

Finally, the review article by Vidana Gamage et al. focusses on an ornamental plant, *Clitoria ternatea*, commonly known as butterfly pea or blue pea flower. The blue pea flower is edible and consumed in the Southeast Asia. Polyacylated anthocyanins known as ternatins contribute to the blue color of the flower. The biosynthesis of ternatins, extraction, stability and antioxidant activity of the anthocyanins were examined. For food application, hot water extraction was recommended and the impact of various extraction conditions on the extraction efficiency and antioxidant activity of the anthocyanins were discussed. The authors also compare the anthocyanins from blue pea flower as natural blue coloring agent with spirulina or phycocyanin and genipin-derived pigments.

This editorial summarizes the articles in the Research Topic *Phytochemicals in Ornamental Plants: Synthesis, Nutraceutical Prospects and Applied Focus*. We hope that this collection of articles will help to expand the basic knowledge on pigment synthesis, plastid biogenesis to the composition of specific bioactive compounds associated with various applications that have great breeding and horticultural importance and confer ornamental plants not only shape, color and beauty but also important nutraceutical potential for the future. Finally, we would like to thank all authors, reviewers and the Frontiers editorial team for their contributions to this Research Topic.

## Author contributions

All authors listed have made a substantial, direct, and intellectual contribution to the work and approved it for publication.

## Conflict of interest

The authors declare that the research was conducted in the absence of any commercial or financial relationships that could be construed as a potential conflict of interest.

## Publisher's note

All claims expressed in this article are solely those of the authors and do not necessarily represent those of their affiliated organizations, or those of the publisher, the editors and the reviewers. Any product that may be evaluated in this article, or claim that may be made by its manufacturer, is not guaranteed or endorsed by the publisher.

